# Immune-mediated inflammatory diseases (IMIDs) in children: key research questions and some answers

**DOI:** 10.1186/s40348-024-00177-7

**Published:** 2024-06-05

**Authors:** Tilmann Kallinich, Marcus A. Mall

**Affiliations:** 1 Department of Pediatric Respiratory Medicine, Immunology and Critical Care Medicine, Charité - Universitätsmedizin Berlin, Berlin, Germany; 2 Deutsches Rheuma-Forschungszentrum (DRFZ) Berlin, Institute of the Leibniz Association, Berlin, Germany; 3 German Center for Lung Research (DZL), associated partner site, Berlin, Germany; 4 Berlin Institute of Health at Charité – Universitätsmedizin Berlin, Berlin, Germany

In children and adolescents, immune-mediated inflammatory diseases (IMIDs) constitute a large spectrum of chronic disorders characterized by complex pathophysiology, substantial disease burden, and often a lack of specific therapies.

In pediatric IMIDs, the inflammation is driven by on-going immunological mechanisms that constantly disrupt immune homeostasis favoring proinflammatory processes (e.g. proinflammatory cytokines, activated T cells and macrophages) while weakening regulatory signals (e.g. interleukin-10, regulatory T and B cells) [1]. Clinically, this leads to acute inflammatory symptoms across various organs and eventually progressive tissue damage and organ dysfunction. Depending on the predominantly affected organs and the extent of systemic inflammation, quality of life and the social participation can be significantly impaired and long-term sequelae including hypertension, dyslipidemia, cardiovascular diseases, chronic kidney disease, cancer, depression, muscular hypotrophy and osteoporosis can develop [2].

The factors driving IMIDs in children continue to be a subject of ongoing research. Increasing prevalence over time, as well as the significant differences in prevalence across the world suggest an important role of environmental factor such as the degree of physical activity, the rate of acute and chronic infections, dietary habits, microbiome dysbiosis, social and cultural changes as well as the exposure to environmental stimuli including industrial toxicants and climate change [2]. In addition, obesity [3], a low exposure to microbes (`hygiene hypothesis´) [4] and stress [5, 6] are associated with the upregulation of the immune response and the development of systemic chronic inflammation in children. Furthermore, maternal exposure to infections, certain diet or stress leads to intrauterine epigenetic changes of the fetus. It has been suggested that these early determinants lead to an intergenerational transmission of certain disease risks including chronic inflammatory processes [2, 7].

Another potential explanation for the increase in IMIDs in children is currently a matter of debate: during early settlements, a tremendous increase in infectious diseases led to genetic adaptations affecting genes related to enhanced host response and protection against threatening pathogen. This hypothesis suggests that the selective pressure during the post-Neolithic period created genetic variants and/or risk factors leading to chronic inflammatory disorders [8]. Since these genetic variations can have deleterious effects on gene function, the associated disease usually manifests in (early) childhood. Familial Mediterranean Fever (FMF) represents a prominent example for this model of disease genesis: mutations within the *MEFV* gene arose through the selective pressure during the plague epidemic [9]. These mutations confer heightened resistance to Yersinia pestis by a reduced binding of the Y. pestis virulence factor YopM. However, they are also associated with FMF (homozygous or compound heterozygous status) and other inflammatory disorders (heterozygous status).

Multifactorial IMIDs such as pediatric systemic lupus erythematosus (pSLE), juvenile dermatomyositis, and systemic juvenile idiopathic arthritis (sJIA, also Still´s disease) are classified based on clinical symptoms and the presence of certain serological biomarkers (e.g. auto-antibodies and S100 proteins, e.g. MRP8/14). Disease differentiation can be challenging in clinical practice due to overlapping symptoms. Additionally, even within one disease, the kind of organ presentation can considerably differ between patients and is often not well described (e.g. involvement of central nervous systeme in pSLE). Moreover, individual responses to therapeutic interventions may vary within a disease group and the same anti-inflammatory therapy may prove effective in patients suffering from different IMIDs. Therefore, defining the individual endotype that describes the underlying pathophysiology is crucial for predicting diseases prognosis and the realization of effective personalized therapy.

This special issue on “Chronic inflammation in Childhood – New Aspects of Mechanisms and Management” was initiated to address important research questions and provide some answers related to various IMIDs with high unmet medical needs. Topics covered include a broad spectrum of IMIDs ranging from sJIA and Familial Mediterranean Fever (FMF) to pSLE, Autoimmune LymphoProliferative ImmunoDeficiency (ALPID) and common complex diseases such as asthma and adipositas. Collectively, the articles included in this collection provide important examples highlighting current progress in the understanding of pathogenesis, improvement in diagnostics, elucidation of different endotypes and development of novel therapeutic strategies for these diseases.

The myeloid-related proteins 8/14 (MRP8/14, also known as S100A8/14 or calprotectin) serve as well-established biomarkers for the diagnosis of sJIA and Familial Mediterranean Fever (FMF). In the article by Dirk Foell et al. a particle-enhanced immune-turbidmetric assay (sCAL turbo) for the routinely detection of MRP8/14 was evaluated in a large patient cohort [10]. The work provides a basis for offering the analysis of MRP8/14 more frequently in routine practice, facilitating quicker and more accurate patient diagnoses.

In FMF it was previously understood that cultured neutrophils from FMF patients spontaneously secret S100A12, Interleukin-18 and caspase-1 [11]. The study by Georg Varga et al. address how this release is mediated. It is shown that neutrophils from patients with FMF are pre-activated by the mutated pyrin (encoded by the *MEFV* gene) and exhibit intrinsically higher levels of reactive oxygen species (ROS) production, leading to increased gasdermin D cleavage [12]. Consequently, soluble mediators are released and the neutrophil undergoes NETosis. These findings highlight gasdermin D as a potential therapeutic target in FMF.

pSLE is characterized by a multifaceted molecular pathophysiology and manifests with multiple phenotypes. The work by Valentina Natoli et al. is dedicated to a rare and poorly described presentation of pSLE [13]. Their comprehensive review proposes two subgroups of neuropsychiatric SLE: (i) a chronic progressive, predominantly type 1 interferon-driven form that poorly responds to currently used treatments, and (ii) an acutely aggressive form that usually presents early during the disease that may be primarily mediated by auto-reactive effector lymphocytes. This work set up the ground for a collaborative discussion across multiple centers to delineate disease subgroups and recommend potential therapeutic interventions.

Due to an impaired immune homeostasis, a subset of inborn errors of immunity (IEI) present with both, signs of immunodeficiency and immune dysregulation. Vasil Toskov and Stephan Ehl elucidate the clinical heterogeneity, pathophysiology, and the challenges in management of five prominent disorders characterized by an “Autoimmune LymphoProliferative ImmunoDeficiency” phenotype (ALPID) [14]. The authors underscore the potential role of somatic mutations in providing proliferative advantages of lymphocytes, contributing to the complexity of the ALPID phenotype. Diagnosing these non-Mendelian mutations requires deeper sequencing techniques.

In asthma bronchiale, inflammatory B cells secret IgE upon allergen contact, thereby playing a pivotal role in the initiation and perpetuation of inflammation. The work by Caroline Kliem and Bianca Schaub describes in detail the different regulatory B cell subsets, which regulate inflammation mainly by expression of the anti-inflammatory cytokines IL-10, TGF-β and IL-35 [15]. This review sheds light on studies in both mice and human (across all age groups) that demonstrate an altered homeostasis of Bregs in asthma, emphasizing their crucial role controlling allergic inflammation.

The concept of delineating endotypes is most thoroughly explored in the context of bronchial asthma. The article by Francesco Foppiano and Bianca Schaub describe the definition of four different inflammatory endotypes: T2-high (eosinophilic), neutrophilic, pauci-granulocytic, and mixed granulocytic asthma [16]. Additionally, initial studies are focusing on developing molecular endotypes utilizing high-throughput technologies such as transcriptomics and mass spectrometry. However, the authors emphasize the limited understanding of the temporal stability of these endotypes. To address this, longitudinal studies are warranted, along with standardization of cluster variables to facilitate cross-study comparisons and enable meta-analyses.

In their article, Lisa Ruck et al. illustrate the complex interplay between chronic inflammation of adipose tissue and the hypothalamus, and the development of obesity [17]. They emphasize the contrasting roles played by central appetite regulators such as leptin and α-melanocyte-stimulating hormone (α-MSH) in the regulation of inflammation. The article proposes hypotheses elucidating the development of local adipose tissue inflammation and its potential systemic spread, which is associated with the development of atherosclerotic cardiovascular disease. Additionally, data illustrating the connection between gut inflammation, hypothalamic inflammation, and obesity development are summarized. The authors discuss the potential of targeting inflammation in adipose tissue and the hypothalamus to mitigate diet-induced obesity and insulin resistance.

This compilation of articles sheds light on recent insights in the understanding disease mechanisms, clinical phenotypes and improvement in diagnostics of IMIDs in children and adolescents. In the forthcoming years, it is anticipated that multi-omics technologies in combination with integrated analysis of big data sets obtained from patient cohorts with different IMID phenotypes will enable a more precise delineation of IMID endotypes. This development on the horizon has the potential to facilitate a more accurate molecular diagnosis, improve prognostic predictions and realize the full potential of precision medicine for children and adolescents with IMDs and high unmet need.
